# Evaluation of the Manchester triage system for patients with acute coronary syndrome

**DOI:** 10.1007/s00508-020-01632-x

**Published:** 2020-04-02

**Authors:** Daniel Kiblboeck, Klara Steinrueck, Christian Nitsche, Wolfgang Lang, Joerg Kellermair, Hermann Blessberger, Clemens Steinwender, Peter Siostrzonek

**Affiliations:** 1 Department of Cardiology and Medical Intensive Care, Med Campus III, Kepler University Hospital, Medical Faculty of Johannes Kepler University, Krankenhausstraße 9, 4021 Linz, Austria; 2 Department of Internal Medicine II - Cardiology, Krankenhaus der Barmherzigen Schwestern, Linz, Austria; 3 Department of Internal Medicine II, Medical University of Vienna, Vienna, Austria

**Keywords:** Manchester triage system, Emergency medicine, Coronary artery disease, Myocardial infarction, Chest pain

## Abstract

**Background:**

An early diagnosis of acute coronary syndrome (ACS) is crucial for treatment and prognosis. The aim of this study was to evaluate the Manchester triage system (MTS) for patients with ACS, e.g. ST-segment elevation myocardial infarction (STEMI), non-ST elevation myocardial infarction (N-STEMI) and unstable angina pectoris (UAP).

**Methods:**

Retrospective analysis of patients diagnosed with ACS (STEMI, N‑STEMI and UAP) who were triaged in the emergency department (ED) with the MTS.

**Results:**

In this study 282 patients with ACS (STEMI: 34.0%, N‑STEMI: 61.7%, UAP: 4.3%) were triaged as MTS level 1 (immediate assessment): 0.4%, MTS level 2 (very urgent): 51.4%, MTS level 3 (urgent): 41.5%, MTS level 4 (standard): 6.7%, MTS level 5 (non-urgent): 0%. We observed significantly lower mean MTS levels in males (male: 2.48 ± 0.59, female: 2.68 ± 0.68, *p* = 0.02) and in patients younger than 80 years (age <80 years: 2.50 ± 0.61, age ≥80 years: 2.70 ± 0.67, *p* = 0.03). We did not find a significant difference of mean MTS levels in different types of ACS (STEMI: 2.46 ± 0.6, N‑STEMI: 2.59 ± 0.64, STEMI vs N‑STEMI: *p* = 0.11, UAP: 2.67 ± 0.65, STEMI vs UAP: *p* = 0.26) and with respect to diabetes (diabetic: 2.47 ± 0.57, non-diabetic: 2.58 ± 0.65, *p* = 0.13). The in-hospital mortality was 2.5% (MTS level 2: *n* = 3, MTS level 3: *n* = 3, MTS level 4: *n* = 1).

**Conclusion:**

The majority of patients with ACS were classified as MTS levels 2 and 3. There was no significant difference of mean MTS levels in patients with STEMI, NSTEMI and UAP. In order to assure an early diagnosis of STEMI, an electrocardiogram (ECG) should be carried out immediately or at least within 10 min after first medical contact in the ED in all patients suspected for ACS, irrespective of the assigned MTS level.

## Introduction

Chest pain is a frequent complaint in the emergency department (ED). Approximately 5–20% of all ED admissions present with chest pain [1]; however, the diagnosis of acute coronary syndrome (ACS) is only confirmed in about 10% of patients who present with chest pain in the ED [2, 3]. An early diagnosis of ACS particularly in patients with ST-elevation myocardial infarction (STEMI), is crucial for treatment and prognosis.

The Manchester triage system (MTS), which was developed by the Manchester Triage Group in 1994, classifies patients based on their main symptoms into five different levels of urgency in terms of the need for first medical assessment (‘MTS level 1 = immediate assessment’, ‘MTS level 2 = very urgent assessment within 10 min’, ‘MTS level 3 = urgent assessment within 30 min [German version]/60 min [British version]’, ‘MTS level 4 = standard assessment within 90 min [German version]/120 min [British version]’, ‘MTS level 5 = non-urgent assessment within 120 min [German version]/240 min [British version]’), irrespective of the eventual diagnosis [4]. Therefore, the MTS uses 52 different flow chart diagrams, e.g. chest pain, abdominal pain, with defined key discriminators, such as danger to life, respiratory distress or state of consciousness to determine the level of urgency for assessment [4].

There is limited data whether there is an influence of MTS levels on treatment strategies and outcome parameters in patients with ACS. The aim of this study was to evaluate the MTS for patients diagnosed with ACS who present primarily in the ED.

## Methods

Patients were identified retrospectively for eligibility in this study by International Statistical Classification of Diseases (ICD) tracking for ICD diagnosis I21.0 (acute transmural myocardial infarction of anterior wall), ICD I21.1 (acute transmural myocardial infarction of inferior wall), I21.2 (acute transmural myocardial infarction of other sites), I21.3 (acute transmural myocardial infarction of unspecified site), I21.4 (acute subendocardial myocardial infarction), I21.9 (acute myocardial infarction, unspecified). All patients with a confirmed diagnosis of ACS (STEMI, non-ST elevation myocardial infarction (N-STEMI) or unstable angina pectoris (UAP), who presented primarily in the ED over a study period of 12 months, were enrolled in this retrospective, single center study. The study was conducted at a large general hospital with an ED volume of about 33,000 patient visits per year with a 24/7 cardiac catheterization laboratory on-site. The triage was done by experienced emergency nurses who had attended an education program for the MTS. Data were collected by retrospective chart review.

The primary objective of this study was defined as the distribution of different MTS levels in patients with ACS. The secondary objectives were defined as a prespecified subgroup analysis of the MTS level distribution for gender, diabetic patients, different types of ACS (STEMI, N‑STEMI and UAP) and age younger and older than 80 years. The study design was approved by the local ethics committee and the study was conducted according to the Declaration of Helsinki.

## Statistical analysis

Categorical variables are described as absolute numbers and percentages. Continuous values are presented as means ± standard deviation. Differences between groups involving normally distributed data were analyzed by the unpaired t‑test; those involving not normally distributed data, by the Mann-Whitney U test; and those involving proportions, by the χ^2^-test. A two-sided *p*-value < 0.05 was considered statistically significant. The *p*-value was not adjusted for multiple testing because the retrospective design of this study was only suitable for hypothesis-generating results. All calculations were performed with SPSS statistical software (Version 22, SPSS Inc., Chicago, IL, USA).

## Results

**Fig. 1:**
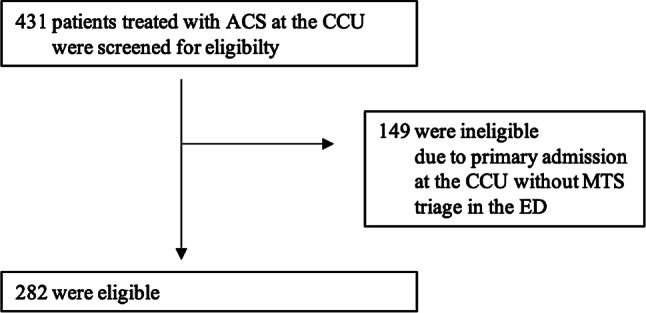
Screening for eligibility in the study. *ACS* acute coronary syndrome; *STEMI* ST-elevation myocardial infarction, *N‑STEMI* non ST-elevation myocardial infarction; *UAP* unstable angina pectoris; *CCU* cardiac care unit; *MTS* Manchester Triage System

During the study period, 431 patients were treated for ACS at this cardiac care unit, 282 of these patients (68.3%) presented primarily in the ED and were triaged with the MTS (Fig. 1). The mean age was 68.0 ± 14.6 years and 90 patients (31.9%) were female. Ninety-six patients (34.0%) presented with STEMI and underwent immediate revascularization in the coronary catheterization laboratory: 174 patients (61.7%) were diagnosed as N‑STEMI and 12 patients (4.3%) as UAP. Coronary angiography was performed in 259 patients (91.8%), while 23 patients (8.2%) were treated conservatively due to severe comorbidities. Overall, 216 patients (76.8%) required percutaneous coronary intervention (STEMI: *n* = 81, 84.4%, N‑STEMI: *n* = 130, 74.7%) and 3 patients were scheduled for coronary artery bypass surgery. The demographic and clinical characteristics of the study population are presented in Table 1.

**Table 1:** Demographic and clinical characteristics of the study population

	All (*n* = 282)	STEMI (*n* = 96)	N‑STEMI (*n* = 174)	UAP (*n* = 12)
Age, years ± SD	68.0 ± 14.6	66.2 ± 15.6	69.4 ± 13.9	61.8 ± 15.5
Age ≥80 years, *n* (%)	61 (21.6)	22 (22.9)	38 (21.8)	1 (8.3)
Female, *n* (%)	90 (31.9)	37 (38.5)	51 (29.3)	2 (16.7)
Hypertension, *n* (%)	197 (69.9)	56 (58.3)	132 (75.9)	9 (75.0)
Diabetes, *n* (%)	90 (31.9)	24 (25.0)	60 (34.5)	6 (50.0)
Smokers, *n* (%)	76 (27.0)	27 (28.1)	45 (25.9)	4 (33.3)
Hyperlipidemia, *n* (%)	170 (60.3)	52 (54.2)	107 (61.5)	11 (91.7)
Prior STEMI, *n* (%)	41 (14.5)	5 (5.2)	30 (17.2)	6 (50.0)
Prior PCI, *n* (%)	58 (20.6)	11 (11.5)	41 (23.6)	6 (50.0)
Prior CABG, *n* (%)	8 (2.8)	1 (1.0)	7 (4.0)	0 (0)
Cardiogenic shock, *n* (%)	7 (2.5)	5 (5.2)	2 (1.1)	0 (0)
PCI, *n* (%)	216 (76.8)	81 (84.4)	130 (74.7)	5 (41.7)
CABG, *n* (%)	3 (1.1)	0 (0)	3 (1.7)	0 (0)
Acetylsalicylic acid, *n* (%)	276 (98.2)	96 (100)	168 (96.6)	12 (100)
Clopidogrel, *n* (%)	71 (25.2)	16 (16.7)	48 (27.6)	7 (58.3)
Prasugrel, *n* (%)	59 (20.9)	51 (53.1)	7 (4.0)	1 (8.3)
Ticagrelor, *n* (%)	148 (52.5)	31 (32.3)	113 (64.9)	4 (33.3)
In-hospital mortality, *n* (%)	7 (2.5)	2 (2.1)	5 (2.9)	0 (0)

*STEMI* ST-elevation myocardial infarction; *N‑STEMI* non ST-elevation myocardial infarction; *UAP* unstable angina pectoris; *PCI* percutaneous coronary intervention; *CABG* coronary artery bypass graft

**Table 2:** Mean MTS levels and standard deviation of the study population and in pre-specified subgroups (different types of acute coronary syndrome, male vs female patients and patients with diabetes vs without diabetes)

	Mean MTS level ± SD	*p*-value
All	2.55 ± 0.63	–
STEMI	2.46 ± 0.6	–
N‑STEMI	2.59 ± 0.64	0.11
UAP	2.57 ± 0.65	0.26
Male	2.48 ± 0.59	–
Female	2.68 ± 0.68	**0.02**
Age <80 years	2.50 ± 0.61	–
Age ≥80 years	2.70 ± 0.67	**0.03**
Diabetes	2.47 ± 0.57	–
No diabetes	2.58 ± 0.65	0.13

*MTS* Manchester Triage System; *STEMI* ST-elevation myocardial infarction; *N‑STEMI* non ST-elevation myocardial infarction; *UAP* unstable angina pectoris

**Fig. 2:**
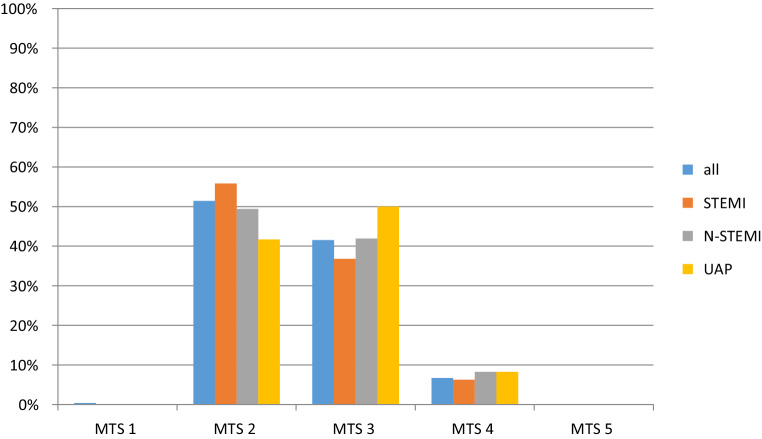
Manchester Triage System (MTS) results of the study population [%]: *blue columns* = all (*n* = 282); *orange columns* = STEMI (*n* = 95); *grey columns* = N‑STEMI (*n* = 172) and *yellow columns* = UAP (*n* = 12). *ACS* acute coronary syndrome; *STEMI* ST-elevation myocardial infarction; *N‑STEMI* non ST-elevation myocardial infarction; *UAP* unstable angina pectoris

The primary objective, which was the distribution of MTS levels in patients with ACS, was 0.4% (*n* = 1) for MTS level 1, 51.4% (*n* = 145) for MTS level 2, 41.5% (*n* = 117) for MTS level 3, 6.7% (*n* = 19) for MTS level 4 and 0% for MTS level 5 (Fig. 2). While 195 patients (69.1%) presented with the chief complaint chest pain (STEMI: *n* = 76, 80.0%, N‑STEMI: *n* = 109, 63.4%, UAP: *n* = 10, 83.3%), 87 patients had other symptoms. Overall, shortness of breath was the second most frequently used flow chart diagram (all: *n* = 32, 11.3%), STEMI: *n* = 5, 5.3%, N‑STEMI: *n* = 24, 14.0%, UAP: *n* = 2, 16.7% followed by the flow chart diagrams unwell adult (all: *n* = 24, 8.5%, STEMI: *n* = 5, 5.3%, N‑STEMI: *n* = 17, 9.9%), palpitations (all: *n* = 8, 2.8%, STEMI: *n* = 1, 1.1%, N‑STEMI: *n* = 7, 4.1%) and collapsed adult (all: *n* = 8, 2.8%; STEMI: *n* = 2, 2.1%; N‑STEMI: *n* = 5, 2.9%) (Fig. 2).

We observed a significant difference between mean MTS levels with respect to gender (male: 2.48 ± 0.59, female: 2.68 ± 0.68, *p* = 0.02) and age (age <80 years: 2.50 ± 0.61, age ≥80 years: 2.70 ± 0.67, *p* = 0.03). There was no significant difference in different types of ACS (STEMI: 2.46 ± 0.6, N‑STEMI: 2.59 ± 0.64, STEMI vs. N‑STEMI: *p* = 0.11, UAP: 2.67 ± 0.65, STEMI vs. UAP: *p* = 0.26) and with respect to diabetes (diabetic: 2.47 ± 0.57, non-diabetic: 2.58 ± 0.65, *p* = 0.13; Table 2).

The documented time of the first ECG was available in 168 patients of our study population. The mean time interval from MTS to ECG was 13.5 ± 17.8 min. There was a shorter mean time interval from MTS to ECG for STEMI patients than for N‑STEMI patients (STEMI: *n* = 62, 9.5 ± 14.2 min, N‑STEMI: *n* = 98, 16.7 ± 19.8 min). The mean time intervals from MTS to ECG were 12 min for MTS level 1 (*n* = 1), 9.0 ± 14.6 min for MTS level 2 (*n* = 89), 19.5 ± 20.8 min for MTS level 3 (*n* = 69), 11.3 ± 8.0 min for MTS level 4 (*n* = 9). Overall, the first ECG was recorded in 115 of the 168 patients (68.5%) within 10 min after MTS (STEMI, *n* = 62: 49 patients [79.0%], N‑STEMI, *n* = 98: 58 patients [59.2%]). The mean maximum values of creatinine kinase as a marker of infarct size were for patients with STEMI or N‑STEMI 1707 U/l (MTS level 1), 891 ± 1229 U/l (MTS level 2), 889 ± 1436 U/l (MTS level 3), and 660 ± 1157 U/l (MTS level 4).

Overall, seven patients had cardiogenic shock at the CCU, two of these presented with cardiogenic shock at the time of triage, five developed this subsequently. Of these patients one was triaged as MTS level 1, four patients as MTS level 2 and two patients as MTS level 4 (Table 3). The in-hospital mortality was 2.5% (*n* = 7), three patients of these patients were triaged as MTS level 2 and 3, respectively and one patient was assigned to MTS level 4.

**Table 3:** Triage results of patients with cardiogenic shock. One patient was triaged as MTS level 1, 4 patients as MTS level 2 and 2 patients as MTS levels 4. Shortness of breath was the most frequently used MTS flowchart. Five of these patients with STEMI had a cardiogenic shock. Overall, 5 patients developed cardiogenic shock after MTS in the ED. The in-hospital mortality of patients with cardiogenic shock was 28.6% (*n* = 2)

ID	MTS level	MTS flow chart	BP at MTS	Type of ACS	Shock onset	Status at hospital discharge
ID28	2	Shortness of breath	105/71	N‑STEMI	After MTS	Alive
ID103	4	Shortness of breath	163/80	N‑STEMI	After MTS	Deceased
ID124	1	Shortness of breath	87/49	STEMI	Before MTS	Alive
ID169	2	Chest pain	108/62	STEMI	After MTS	Alive
ID317	2	Collapsed adult	61/32	STEMI	Before MTS	Alive
ID327	2	Shortness of breath	119/97	STEMI	After MTS	Deceased
ID413	4	Vomiting	134/66	STEMI	After MTS	Alive

*MTS* Manchester Triage System; *BP* blood pressure; *ACS* acute coronary syndrome

## Discussion

Our retrospective study demonstrates that the majority of patients (92.9%) diagnosed with ACS who presented primarily in the ED were triaged as MTS level 2 and MTS level 3 (very urgent to urgent assessment within 10–30 min [German version] or 60 min [British version]). We observed significantly lower MTS levels for males and patients younger than 80 years while there was no significant difference with respect to different types of ACS and patients with diabetes.

Due to an increasing number of patient visits in ED, triage tools become more important in ED to prioritize the assessment and treatment of patients with potentially life-threatening diseases, such as ACS. An early diagnosis of STEMI in the ED is crucial for an early revascularization strategy in the coronary catheterization laboratory.

Patients who present to the ED are classified by the MTS based on their chief complaints into five different levels of urgency for their need of assessment according to 52 different flowchart diagrams, irrespective of the eventual diagnosis [4].

The recommended target time for an ECG is less than 10 min after first medical contact for patients with ACS according to current guidelines in our study population [5]. Data about the documented time of the first ECG after MTS was available in 168 of our study population. Therefore, the presented results need to be interpreted with caution. However, we detected a shorter mean time interval from MTS to the first ECG for STEMI patients compared to N‑STEMI patients (STEMI: 9.5 ± 14.2 min, N‑STEMI: 16.7 ± 19.8 min). Remarkably, we observed a trend for mean time intervals from MTS to the first ECG MTS with respect to different MTS levels (MTS 1 (*n* = 1): 12 min, MTS 2 (*n* = 89): 9.0 ± 14.6 min, MTS 3 (*n* = 69): 19.5 ± 20.8 min, MTS 4 (*n* = 9): 11.3 ± 8.0 min). The first ECG was written in 68.5% (*n* = 115) of patients with ACS, 79.0% of STEMI patients (*n* = 49) and 59.2% of NSTEMI patients (*n* = 58) within 10 min after MTS. Gouvea et al. reported a mean time interval from MTS to ECG for all patients with ACS of 24.9 ± 31.1 min and a mean time interval from MTS to ECG for MTS level 1 and 2 patients of 19.46 ± 24.62 min [6]. Therefore, we consider that in patients with suspected ACS it is very important to write an ECG immediately or at least within 10 min after first medical contact in the ED irrespective of the assigned MTS level, in order to assure an early diagnosis of STEMI in this high-risk population. Short door-to-balloon times are important for prognosis in patients with STEMI. We observed a trend for higher mean maximum values of creatinine kinase as a marker of infarct size for patients triaged as MTS 1–3 (MTS 1: 1707 U/l, MTS 2: 891 ± 1229 U/l, MTS 3: 889 ± 1436 U/l, MTS 4: 660 ± 1157 U/l). However, we consider that maximum values of creatinine kinase were more influenced by the time from symptom onset to revascularization than by the assigned MTS levels.

Matias et al. demonstrated a comparable MTS level distribution to our study in a smaller study population of 114 patients with ACS (MTS level 1: 0.9%, MTS level 2: 62.3%, MTS level 3: 16.7%, MTS level 4: 10.5%, MTS level 5: 1.8%) [7]. The relatively low proportion of patients classified as MTS level 1 in this and our study might be explained by direct admissions to medical intensive care units of hemodynamically and/or respiratory unstable patients bypassing the ED.

The sensitivity of the MTS for detecting high risk cardiac patients by nurses was reported to be 86.8% (95% CI 78.4–92.3%) with a specificity of 72.4% (95% CI 61.4–81.2%) [8]. A slightly higher sensitivity of 87.3% (95% CI 83.1–90.6%) was reported for the MTS in assigning high priority (MTS level 1 and 2) to patients with ACS [9].

**Fig. 3:**
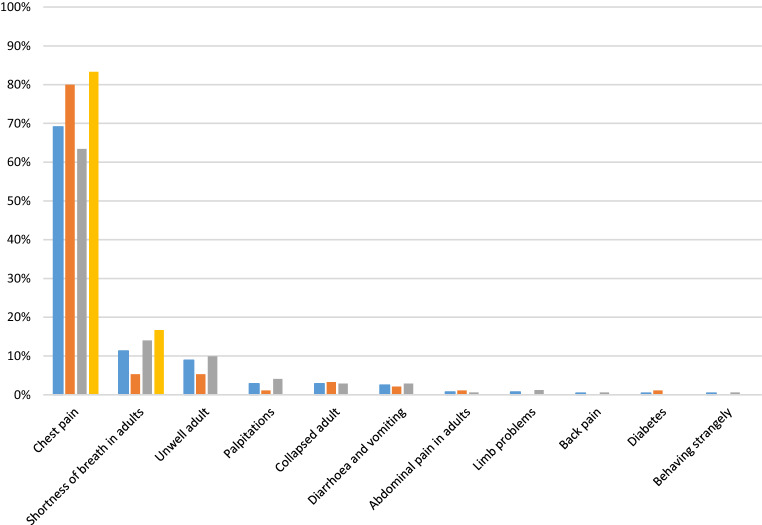
Flow chart diagrams used for patients with ACS: blue columns = ‘all (*n* = 282)’, orange columns = ‘STEMI (*n* = 95)’, grey columns = ‘N-STEMI (*n* = 172)’ and yellow columns = ‘UAP (*n* = 12)’. Chest pain was the most frequently used flowchart diagram (all: 69.1%, STEMI: 80.0%, N‑STEMI: 63.4%, UAP: 83.3%) followed by shortness of breath in adults (all: 11.3%, STEMI: 5.3%, N‑STEMI: 14.0%, UAP: 16.7%) and unwell adult (all: 8.9%, STEMI: 5.3%, N‑STEMI: 9.9%). *ACS* acute coronary syndrome; *STEMI* ST-elevation myocardial infarction; *N‑STEMI* non ST-elevation myocardial infarction; *UAP* unstable angina pectoris

We did not observe a significant difference of mean MTS levels with respect to different types of ACS. Therefore, differentiation of STEMI from N‑STEMI or UAP is from our experience not possible with the MTS, as the diagnosis of STEMI, N‑STEMI and UAP is based on ECG criteria and troponin levels. However, it has been proven that the MTS is effective in patients with STEMI with typical symptoms with respect to first medical assessment within target time [10]. The observed higher mean MTS levels for female and patients older than 80 years may be explained by a presentation with other or atypical symptoms. Chest pain was the most frequently used flow chart diagram in our study population (*n* = 195, 69.1%) and was even more frequently used in patients with STEMI (*n* = 76, 80.0%) (Fig. 3). Fewer patients with N‑STEMI (*n* = 109, 63.4%) complained about chest pain and shortness of breath was more frequently in patients with N‑STEMI (*n* = 24, 14.0%) compared to patients with STEMI (*n* = 5, 5.3%). Other flow chart diagrams were less frequently used in our study population (unwell adult: *n* = 24, 8.5%; palpitations: *n* = 8, 2.8%; collapsed adult: *n* = 8, 2.8%). Two hundred and twenty-seven patients (80.5%) presented with typical symptoms of myocardial infarction (chest pain or shortness of breath). Therefore, we believe that the MTS is a valuable tool to detect patients with STEMI or N‑STEMI early in the ED.

Remarkably, our study demonstrated that the majority of patients with cardiogenic shock had an onset of shock after triage in the ED. Therefore, we believe that patients with suspected ACS with typical symptoms, e.g. chest pain, shortness of breath, should be monitored on cardiac care units to detect deteriorating patients early, irrespective of the assigned MTS level. The in-hospital mortality of 2.5% was low. Three of these patients were classified as MTS level 2 and 3, respectively and one patient as MTS level 4. Several studies have shown that the MTS predicts hospital admission and mortality in an unselected all-comers study population [11–14].

No data were found in the literature for the emergency severity index and the Australasian triage scale with respect to acute coronary syndrome or myocardial infarction. A large study in Canada which included over 3000 patients with STEMI and NSTEMI demonstrated that half of the patients were given a low acuity triage score of 3, 4 or 5 with the Canadian triage and acuity scale which was independently associated with substantial delays in ECG acquisition (median door-to-ECG time 12.0 min with a 4.4 min delay in median door-to-ECG time) and to reperfusion therapy (median door-to-needle time 40.0 min with a 15.1 min delay in median door-to-needle time). Therefore, the authors concluded that the quality of ED triage may have an important impact on acute myocardial infarction care [15].

## Limitations of this study

However, we are well aware of the fact that our findings must be interpreted with caution. First, it is a single center experience. Second, the retrospective design can only be considered as hypothesis generating. Third, the number of patients is limited.

## Conclusion

The majority of patients diagnosed with ACS, who presented to the ED and were triaged by the MTS, were classified as MTS levels 2 and 3 (very urgent to urgent assessment within 10 to 30 min [German version]/60 min [British version]). We observed significant lower MTS levels for males and patients younger than 80 years. As discrimination of different types of ACS is not possible with the MTS, we recommend that an ECG should be recorded in patients suspected for ACS immediately or at least within 10 min after first medical contact in the ED according to current guidelines, irrespective of the assigned MTS level in order to assure an early diagnosis of STEMI.
